# Faricimab versus bevacizumab for neovascular age‐related macular degeneration: Cost analysis based on real‐world data from the Swedish Macula Registry

**DOI:** 10.1111/aos.16774

**Published:** 2024-10-14

**Authors:** Souad Abdalla, Inger Westborg, Anni‐Maria Pulkki‐Brännström, Helena Norberg

**Affiliations:** ^1^ Department of Medical and Translational Biology Umeå University Umeå Sweden; ^2^ Department of Clinical Sciences/Ophthalmology Umeå University Umeå Sweden; ^3^ Department of Epidemiology and Global Health Umeå University Umeå Sweden

**Keywords:** AMD, cost minimization analysis, faricimab, real‐world data, Swedish Macula Registry

## Abstract

**Purpose:**

To analyse the impact on cost if faricimab is used as the first‐line treatment for neovascular age‐related macular degeneration (nAMD) compared to standard treatment with bevacizumab.

**Methods:**

Retrospective registry study including real‐world data from the Swedish Macula Registry between 2017 and 2022. The observed number of injections and visits for bevacizumab during the first two years of treatment was used (*n* = 437 patients). Number of faricimab injections was obtained from published clinical trial data and unit costs mostly from publicly available Swedish sources. The provider cost included medication and visit cost and societal cost included additionally patient travel cost. Costs are presented in 2023 EUR.

**Results:**

The incremental societal cost of faricimab was 277 EUR per patient compared to bevacizumab in the base case. Medication cost was higher (1516 EUR) while visit cost (−1183 EUR) and patient travel cost (−56 EUR) were lower due to longer injection intervals. Faricimab was of similar cost as bevacizumab for patients residing far from the clinic. The faricimab injection interval and the number of bevacizumab injections were major drivers of uncertainty in the results.

**Conclusion:**

Faricimab represents a cost‐effective alternative to bevacizumab for patients with nAMD in Sweden. Its extended treatment interval is particularly beneficial for patients living far from clinics, and if the real‐life faricimab injection interval extends beyond 12 weeks. Our findings emphasize faricimab's potential to free up healthcare staff to treat a larger patient population with existing clinic resources.

## 1 INTRODUCTION

Age‐related macular degeneration (AMD) poses a significant threat of vision loss, especially in individuals over 60 years, and impacts over 200 million people globally (Flaxman et al., 2017; Wong et al., 2014). Neovascular AMD (nAMD) is a chronic disease that often requires lifelong treatment, indicating that the prevalence is increasing gradually over time (Chandra et al., 2020; Westborg et al., 2017). Current treatments involve anti‐vascular endothelial growth factor (VEGF) intravitreal injections (Mitchell et al., 2018). Anti‐VEGF agents have transformed the treatment options for nAMD, reducing the incidence of blindness caused by nAMD by half (Bloch et al., 2012; Granstam et al., 2016). The anti‐VEGF agents approved for nAMD are aflibercept, ranibizumab, and brolucizumab. Bevacizumab binds to all forms of VEGF‐A and is often used off‐label due to a significantly lower price compared to other agents. Anti‐VEGF agents require frequent injections, which implies a high burden on patients' and caregivers' quality of life (Boyle et al., 2018; Gopinath et al., 2014; Mitchell & Bradley, 2006; Varano et al., 2015), overloads healthcare, and is costly (Jörstad et al., 2020; Kristiansen et al., 2020; Prenner et al., 2015).

In Sweden, aflibercept was ranked among the top four best‐selling drugs in 2023, with sales exceeding 1 billion Swedish krona (SEK) (Lif, 2023). To manage the high‐cost burden of anti‐VEGF treatment, many Swedish counties introduced bevacizumab as a first‐line treatment (CATT, 2011). In Sweden, several drug therapeutic committees also recommend bevacizumab as the first‐line treatment. Since it is an off‐label drug, few health economic evaluation studies have included bevacizumab as a comparator; however, some studies have found it to be less costly and/or the most cost‐effective option (Brown et al., 2020, 2021; CADTH 2020; Quist et al., 2022).

If disease control could be maintained with less frequent injections, it would be of significant value to both patients and healthcare providers while also reducing costs (Reitan et al., 2023). Studies have shown that the use of bevacizumab, compared to other anti‐VEGF agents requires more frequent injections over the first years (Berg et al., 2015; Park et al., 2017 ). There is a notable desire among both healthcare providers and patients for medications with extended treatment intervals. By extending the treatment intervals, the health care can alleviate its workload, leading to a more cost‐effective use of healthcare resources. Extending the intervals between treatments would also benefit patients as they often find it demanding to attend frequent appointments at the eye clinic (Boyle et al., 2018; Droege et al., 2013; Sivaprasad & Oyetunde, 2016). Less frequent visits would not only reduce the logistical challenges, such as transportation issues and the need for a caregiver to be available for every visit, but would also ease the lives of individuals residing far away from their closest clinic (Prenner et al., 2015).

Faricimab is a new drug that has shown comparable health outcomes with ranibizumab and aflibercept in terms of preservation of vision in phase II and III trials (Heier et al., 2022; Khanani et al., 2020, 2024). Faricimab can be administered with extended treatment intervals up to 16 weeks and showed non‐inferiority compared to aflibercept every 8 weeks in the phase III trials, TENAYA and LUCERNE (Heier et al., 2022; Khanani et al., 2024). Approximately 63% of participants in the faricimab arm were treated every 16 weeks, and nearly 80% received injections every 12 or 16 weeks at week 112 (Khanani et al., 2024). Despite the evidence of the potential usefulness of faricimab, there is currently no health economic evaluation in Sweden for the use of faricimab that could support the implementation of faricimab in clinical practice. The Swedish Dental and Pharmaceutical Benefits Agency (TLV) has conducted health economic evaluations of the current anti‐VEGF agents used in Sweden: aflibercept, ranibizumab and brolucizumab (TLV, 2012, 2013, 2020). These evaluations focused on cost per quality‐adjusted life year (QALY) and were based on the list price of each drug. The list price is the standard retail price set by the pharmaceutical manufacturer before any discounts or negotiations with payers. Because every county in Sweden negotiates price with the company, and it is common to split ampules to several syringes, the list price is not always representative of what the counties pay, which makes the evaluations less applicable in clinical practice. Therefore, a health economic evaluation of faricimab—from both a provider and societal perspective—would offer a more robust foundation for decision‐making to prioritize which primary treatment to use in the clinics, leading to a more cost‐effective approach and improved patient outcomes.

The aim of this study was to analyse how provider and societal costs are affected if the new anti‐VEGF agent faricimab is used as the first‐line treatment for nAMD, comparing it to current standard treatment using bevacizumab in ophthalmology clinical settings.

## 2 METHOD

This study is a cost minimization analysis based on data from the Swedish Macula Registry (SMR).

### 2.1 Data source

#### 2.1.1 Swedish macula registry

SMR is a national quality registry which was established in 2003 and transitioned to a web‐based version in 2008 (SMR, 2015). SMR was established for monitoring the treatment of nAMD and it includes 48 public and private medical facilities (Westborg et al., 2023). SMR includes various variables such as diagnosis, gender, age, distance and near visual acuity, type of macular degeneration, demographics, type of treatment, and treatment frequency. Data reporting to the registry occurs at the initial treatment visit as well as during follow‐up and subsequent treatment visits. The reporting of data is on a voluntary basis for both clinics and patients. In 2022, the registry had a coverage rate of 88%, a steady increase in the number of newly registered patients has been shown. The pandemic likely had an impact on the number of new cases requiring treatment, particularly in the early stages of the COVID−19 pandemic. Since nAMD is a chronic disease, there is an increase in the number of patients each year that requires regular follow‐up visits. As of 31 December 2022, the registry covered a total of 55 205 patients, 68 549 eyes, accounting for a cumulative 1 216 079 visits (including treatment visits). The main purpose of the registry is to achieve consistent national monitoring, quality assurance, and evaluation of nAMD treatment.

### 2.2 Participants

Research participants were drawn from SMR patients registered between the years 2017 and 2022. The participants were selected from three counties: Norrbotten, Västerbotten, and Östergötland. These counties were selected due to the geographic differences in terms of area. Norrbotten county has an area of 97 239 km^2^, Västerbotten has an area of 54 665 km^2^, whereas Östergötland county has an area of 10 000 km^2^. The variation of area allows data fluctuations in the distances patients must travel to reach clinics, spanning both short and long distances to the clinics. In Norrbotten and Västerbotten counties there are three clinics where the patients receive injections. In Östergötland county the patients can receive injections at two clinics, but only patients from one clinic were included in this analysis due to the other clinic's lower SMR coverage rate.

The participants were selected by applying the following criteria: diagnosis nAMD registered between 2017 and 2022, aged 50 and above, treatment‐naive eyes, bevacizumab treatment only. Additionally, participants were only included if having data registered for two complete years within an interval of plus or minus two months. Participants needed to have received at least three injections during the first year and at least one injection during the second year. Exclusion criteria were participants who had undergone previous treatment with anti‐VEGF, laser, or photodynamic therapy (PDT), as well as those who had received bilateral treatment.

### 2.3 Treatment strategies

The comparators in this analysis are bevacizumab and faricimab. Bevacizumab and ranibizumab have shown similar effects in vision preservation (CATT, 2011). Faricimab has shown comparable visual benefits with both ranibizumab and aflibercept (Heier et al., 2022; Khanani et al., 2020, 2024). No previous study has directly compared bevacizumab and faricimab. We therefore assumed that faricimab has at least similar effects in relation to bevacizumab for vision preservation.

Both treatments are humanized monoclonal antibodies that inhibit the activity of VEGF receptors, improving visual acuity by preventing abnormal blood vessel growth, decreasing vascular permeability, and increasing vascular stability (Panos et al., 2023). Faricimab is a bispecific antibody that in addition to VEGF also inhibits angiopoietin‐2. Faricimab allows for extended treatment intervals of up to 16 weeks and demonstrated comparable efficacy to aflibercept administered every 8 weeks in the TENAYA and LUCERNE trials (Heier et al., 2022; Khanani et al., 2024). Study participants initially received four monthly faricimab intravitreal injections, after which the frequency of treatment was adjusted to intervals of 8, 12, or 16 weeks depending on disease activity. Roughly 45% of participants receiving faricimab were treated every 16 weeks, with nearly 80% receiving injections at intervals of either 12 or 16 weeks in week 48 (Heier et al., 2022). In year 2, the study protocol allowed extension of treatment intervals. Consequently, in week 112, the proportion receiving injections every 16 weeks had increased to 63%, while the number receiving injections every 12 to 16 weeks was still about 80%. (Khanani et al., 2024). In comparison, bevacizumab is typically administered with three monthly intravitreal injections, followed by intervals of 4, 8 or sometimes 12 weeks depending on the level of disease activity.

The use of bevacizumab for off‐label nAMD treatment in Sweden is associated with the lowest cost per injection compared to other available anti‐VEGF agents. Data from SMR for the three counties indicates a significant preference for bevacizumab over aflibercept and ranibizumab in terms of injection administration. However, due to the recent regulatory approval of faricimab, no registrations of faricimab were available in SMR at the time of this analysis. Some clinics began incorporating faricimab into their treatment protocols towards the end of 2023, after the study period of the current analysis. Consequently, the analysis was conducted based on patients treated with bevacizumab within the SMR database.

### 2.4 Cost analysis

The direct costs included in the comparison are as follows:

1. the cost of the medication
2. the cost of the administration, including visit to nurses or physicians including the operation cost, the materials costs such as syringes, and compounding cost
3. the cost of patients' travels to clinics

For bevacizumab and faricimab, costs were assessed during the first two years after treatment initiation. First from a provider perspective by including direct healthcare costs of the visits where the patient received an injection, and second from a societal perspective including additionally direct patient costs for travel. The visits where the patient did not receive an injection were excluded from the analysis because these visits were assumed to be unaffected by treatment strategy.

Patient‐level total costs were calculated by multiplying the number of visits (injection count) with the unit costs. For bevacizumab, the injection count was the number observed in the data. The count was computed for the first year (0–365 days) and the second year (366–730 days) separately before adding up. For faricimab, a base case injection count was constructed that describes the likely implementation in clinical practice if faricimab was used as first‐line treatment. The base case assumption about faricimab was taken from the TENAYA and LUCERNE trials (Heier et al., 2022). The base case number of faricimab injections was 6.94 injections during the first year and 4.2 injections during the second year. Notably, this base case scenario is close to a 12‐week interval after the three months of bolus injections (7 injections first year, 4 injections second year).

The price of the anti‐VEGF medications is the result of negotiations between each healthcare region and the pharmaceutical company. The cost per injection depends on the negotiated price and on how many injections are compounded from each ampule. In practice, the prices are confidential, and the injections are often compounded by the hospital pharmacy. The price of bevacizumab was provided by one region in Sweden. The price of faricimab was assumed to be equal to aflibercept. The cost per injection was calculated assuming that the injections are dispensed by the hospital pharmacy. Each medication ampule was considered to be dispensed into 20 injections for bevacizumab and into three injections for faricimab. No‐residual syringes and needles are used for faricimab to maximize the number of injections from each ampule. These syringes and needles are more expensive than standard. The visit cost was collected from the Northern Healthcare Regional Association pricelists (Northern Healthcare Regional pricelist, 2023) assumed to be equal for both treatment strategies. The cost was confirmed by one clinic. Unit costs are presented in Table 1.

**TABLE 1:** Unit costs of the alternative treatment strategies, in 2023 Euros.

	Bevacizumab	Faricimab
Medication cost (EUR/injection)	27	172
Price of medication	14	131
Hospital pharmacy compounding	13	13
Syringe & needle	0.17	28
Visit cost (EUR/injection)	331	331
Patients' travel cost (EUR/km)	0.22	0.22

*Note*: Syringes and needles used for pharmacy compounded faricimab are non‐residual and therefore more expensive than standard syringes and needles.

The direct patients' costs included travel expenses for patients. This was calculated based on the distance between the participants' municipality and their clinic. Information about the clinic for each visit was available in the dataset. Participants' municipality was available but not their specific residential location. Therefore, the distance to the municipality city centre was used. Distance was multiplied by two to account for a return trip per visit. Patients' travel cost was assigned a fixed price per kilometre equivalent to the payroll tax reduction rate for travelling with own car (Swedish Tax Agency, 2023). Thus, the total patient travel cost was calculated as follows:

$$\begin{array}{c} \text{Patients}’\;\text{travel cost}\\ \;=\text{injection count}\;*\;\text{distance in}\;\mathrm{km}\;*\;2\;*\;0.22\;\mathrm{EUR}/\mathrm{km}. \end{array}$$

Costs were collected and analysed in Swedish krona (SEK) and converted to Euros using the 2023 average exchange rate 1 EUR = 11.4788 SEK. No discounting was applied to adjust costs to present values because the time period was short.

### 2.5 Subgroup analysis

The subgroup was selected to capture those with the longest travel distance, that is residing far away from the clinic. No patients had a travel distance between 34 and 54 km and in the region with the longest distances, the mean travel distance was 47 km. Therefore, we pragmatically selected 50 km as a cut‐off to define the subgroup with the longest travel distance. The same cost calculations as for the full study population were then applied to this subgroup.

### 2.6 Sensitivity analysis

A one‐way deterministic sensitivity analysis was performed to investigate how sensitive the results are to uncertainty in assumptions and variation in unit costs (Drummond, 2015). First, we varied the interval between faricimab injections, which were taken from the TENAYA and LUCERNE trials in the base case. In the sensitivity analysis, the interval was varied from 16 weeks (equivalent to 6 injections during the first year and 3 injections during the second year) to 8 weeks (equivalent to 8 injections during the first year and 6 injections during the second year).

One exclusion criterion in our study was switch to another anti‐VEGF. A large group of patients (*n* = 437) were excluded. The excluded patients probably had a more active disease and inadequate response to bevacizumab. The study population had fewer injections (14.7) over two years compared with the Lucas study in Norway where the patients had 18.2 injections (Berg et al., 2015). We therefore added a sensitivity analysis with 18 injections of bevacizumab for each patient over two years.

A 20% reduction in the price of medication was used to investigate the potential consequences of regions' price negotiations with the manufacturer. The unit cost of a physician or nurse visit, for which an average cost from Northern Healthcare Regional pricelist was used in the base case, was varied up and down around the base case value.

Finally, a four‐fold increase in the unit cost of travel was considered to reflect the fact that most patients cannot travel to the clinic by car on their own and/or are accompanied by another person due to the nature of the treatment.

### 2.7 Ethical approval

The study was approved by the Swedish Ethical Review Authority (Dnr 2023‐02738‐01) and complies with the Declaration of Helsinki. At the start of treatment for nAMD, patients are informed of the SMR and that they have the right to opt out of the register at any time. Patients receive comprehensive information about the SMR, the intended use of their information for purposes including quality assurance in healthcare, statistical analysis, and ethically approved research. The extracted SMR data was pseudonymized. Research data was managed according to Umeå University regulations for secure data management.

## 3 RESULTS

**FIGURE 1:**
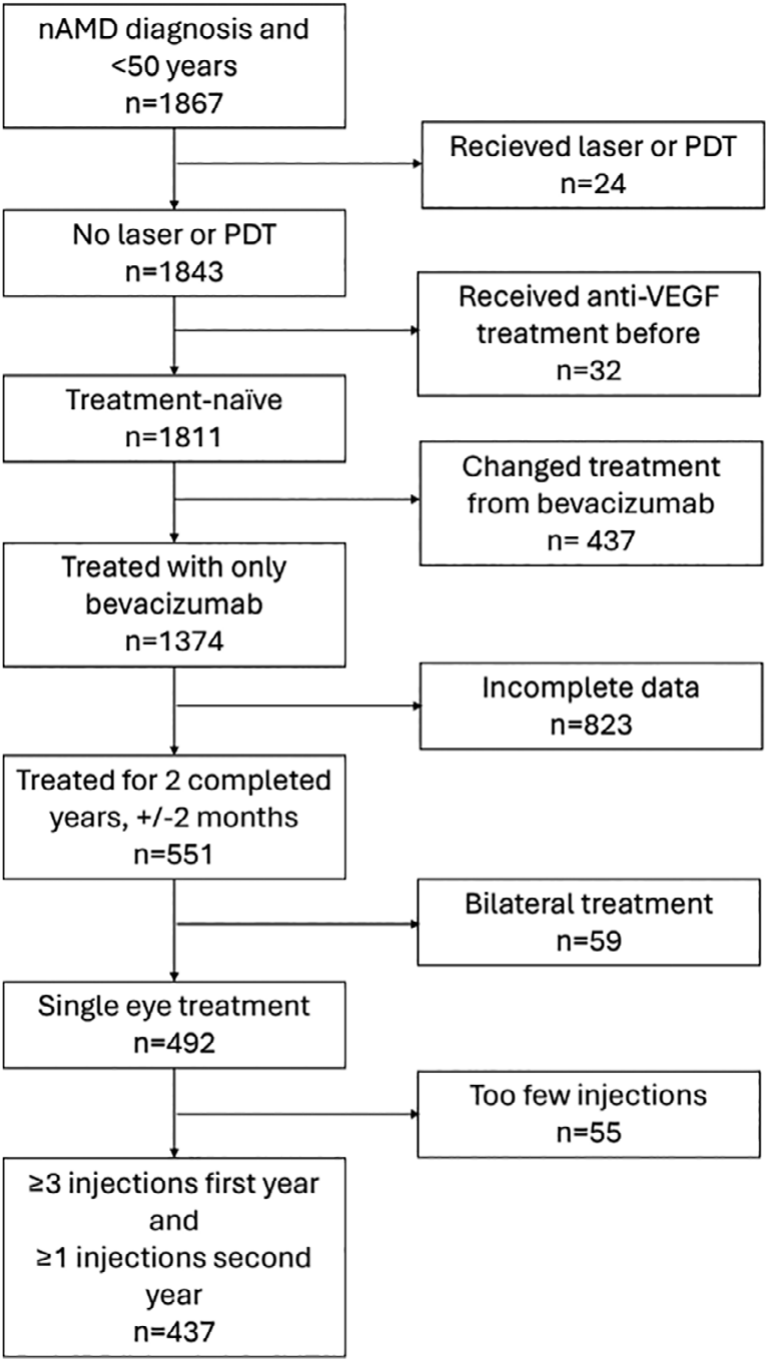
Flowchart illustrating the effect on participant numbers of the application of the selection criteria.

According to SMR there were 1867 registered patients with nAMD between 2017 and 2022 in Norrbotten, Västerbotten and Östergötland counties. Figure 1 illustrates the selection of patients based on the established criteria. Out of the 1867 patients, 1843 had not received laser or PDT. Of these, 1811 patients were treatment‐naïve for anti‐VEGF medications, and 1374 patients had only been treated with bevacizumab. After excluding patients with incomplete data (*n* = 823), those who received bilateral treatment (*n* = 59), and those who received fewer than 3 injections in the first year and no injections in the second year (*n* = 55), a total of 437 (23%) patients remained who met all the selection criteria.

### 3.1 Travel distance

**FIGURE 2:**
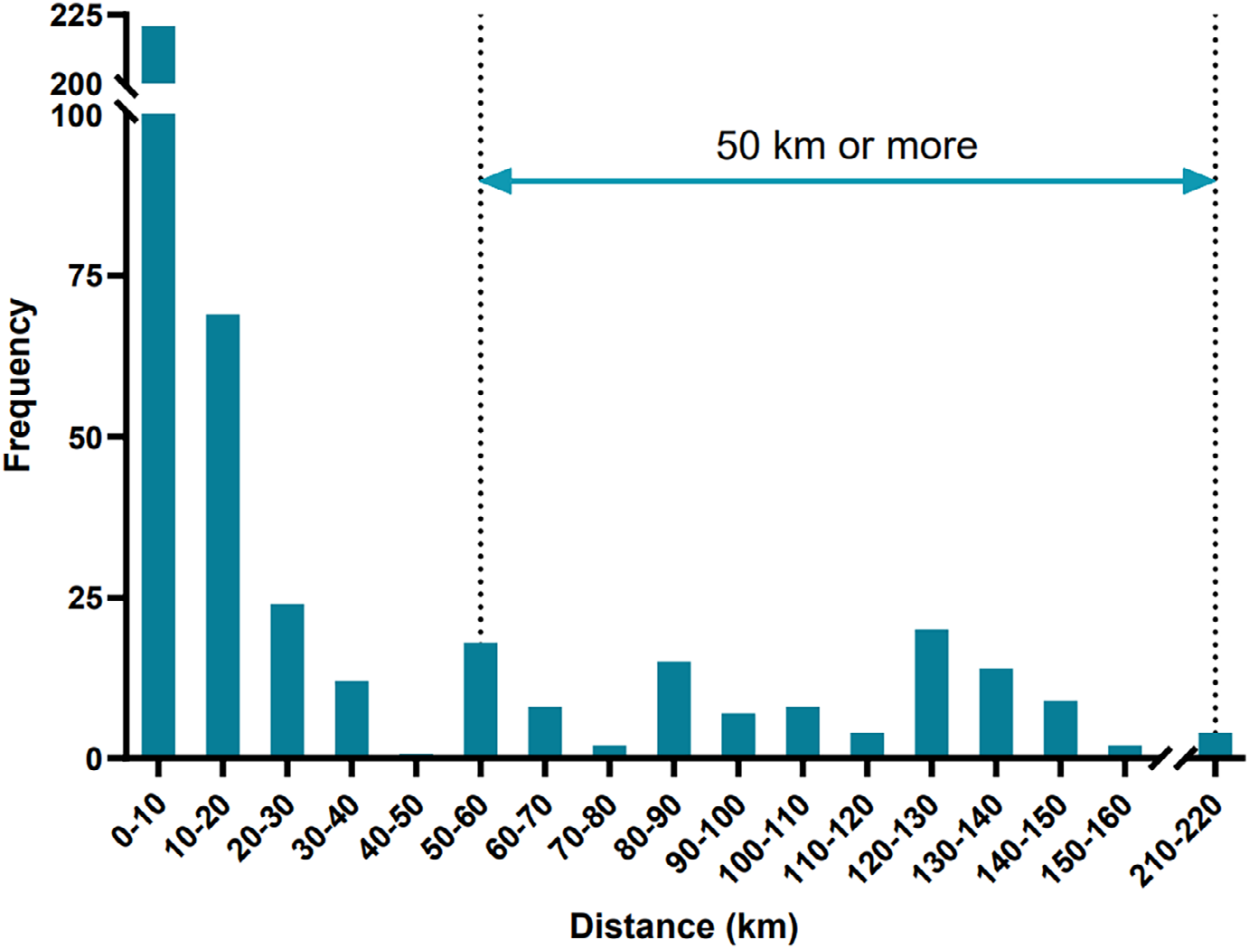
The distribution of one‐way distances in kilometres to the eye clinic in the counties of Norrbotten, Västerbotten and Östergötland.

There was notable variation in travel distances for patients to reach the clinic as shown in Figure 2. In addition, there were notable variations in mean travel distance between the three regions of Norrbotten, Västerbotten, and Östergötland. In Norrbotten, the one‐way mean travel distance was 47 km (range 1.9–158 km), in Västerbotten it was 22 km (range 0.6–218 km) and in Östergötland it was 10 km (range 3.5–88 km).

### 3.2 Average cost per patient

The average number of bevacizumab injections over the course of the two‐year study period in the study population was 14.7. The mean total travel distance during the same period was calculated to be 978 km. In the base case for faricimab, all patients were assumed to receive the same number of injections, 11.1 (6.94 in year 1 and 4.2 in year 2). The mean total travel distance with faricimab was calculated to 721 km. Table 2 shows the average provider cost, patient travel cost, and societal cost per patient for current practice, faricimab, and the difference between these for the study population (*n* = 437). From the provider perspective, faricimab costs 333 EUR more per patient than current practice over the two‐year treatment period. The medication cost of faricimab is higher by 1516 EUR, yet there is a substantial saving of 1183 EUR in the visit cost. For patients, faricimab brings a cost saving of 56 EUR arising from fewer clinic visits. From the societal perspective, faricimab costs 277 EUR more per patient over two years compared to current practice. Table 3 presents a breakdown of these costs for the first and second years. The visit cost is lower for faricimab after the first year, and during the second year, faricimab is cost saving overall (−173 EUR) even if medication cost still is higher.

**TABLE 2:** Average costs per patient in 2023 Euros comparing current practice and faricimab as a first‐line treatment over two years.

	Current practice	Faricimab	Difference
Provider cost	5276	5600	333
Medication cost	396	1912	1516
Visit cost	4871	3688	−1183
Patient travel cost	213	157	−56
Societal cost Sum of previous	5480	5757	277

**TABLE 3:** Average cost per patient in 2023 Euros comparing current practice and faricimab as a first‐line treatment specified as first and second years.

	First year			Second year		
	Current practice	Faricimab	Difference	Current practice	Faricimab	Difference
Provider cost	3019	3498	470	2249	2111	−137
Medication cost	227	1191	964	169	721	552
Visit cost	2791	2297	−494	2079	1390	−689
Patient travel cost	118	98	−20	95	59	−36
Societal cost Sum of previous	3136	3586	450	2344	2170	−173

### 3.3 Subgroup analysis

The number of patients travelling at least 50 km to reach the clinic was 111 patients, which represents 25% of the study population (*n* = 437). The average number of injections in this subgroup was 14.9 injections over the course of the two‐year study period, which was close to the mean (14.7) in the study population as a whole. The mean travel distance was 3130 km for current practice and 2344 km for faricimab. Table 4 shows a minimal difference in the societal cost per patient at 104 EUR per patient over two years. In this subgroup, the higher medication cost of faricimab (1511 EUR) is cancelled out by savings to the provider from visit cost (−1236 EUR) and savings to patients from travel (−171 EUR).

**TABLE 4:** Overview of the average cost per patient in 2023 Euros for participants travelling at least 50 km as a one‐way trip to the clinic.

	Current practice	Faricimab	Difference
Provider cost	5324	5600	275
Medication cost	400	1912	1511
Visit cost	4924	3688	−1236
Patient travel cost	682	511	−171
Societal cost Sum of previous	6006	6110	104

### 3.4 Sensitivity analysis

**FIGURE 3:**
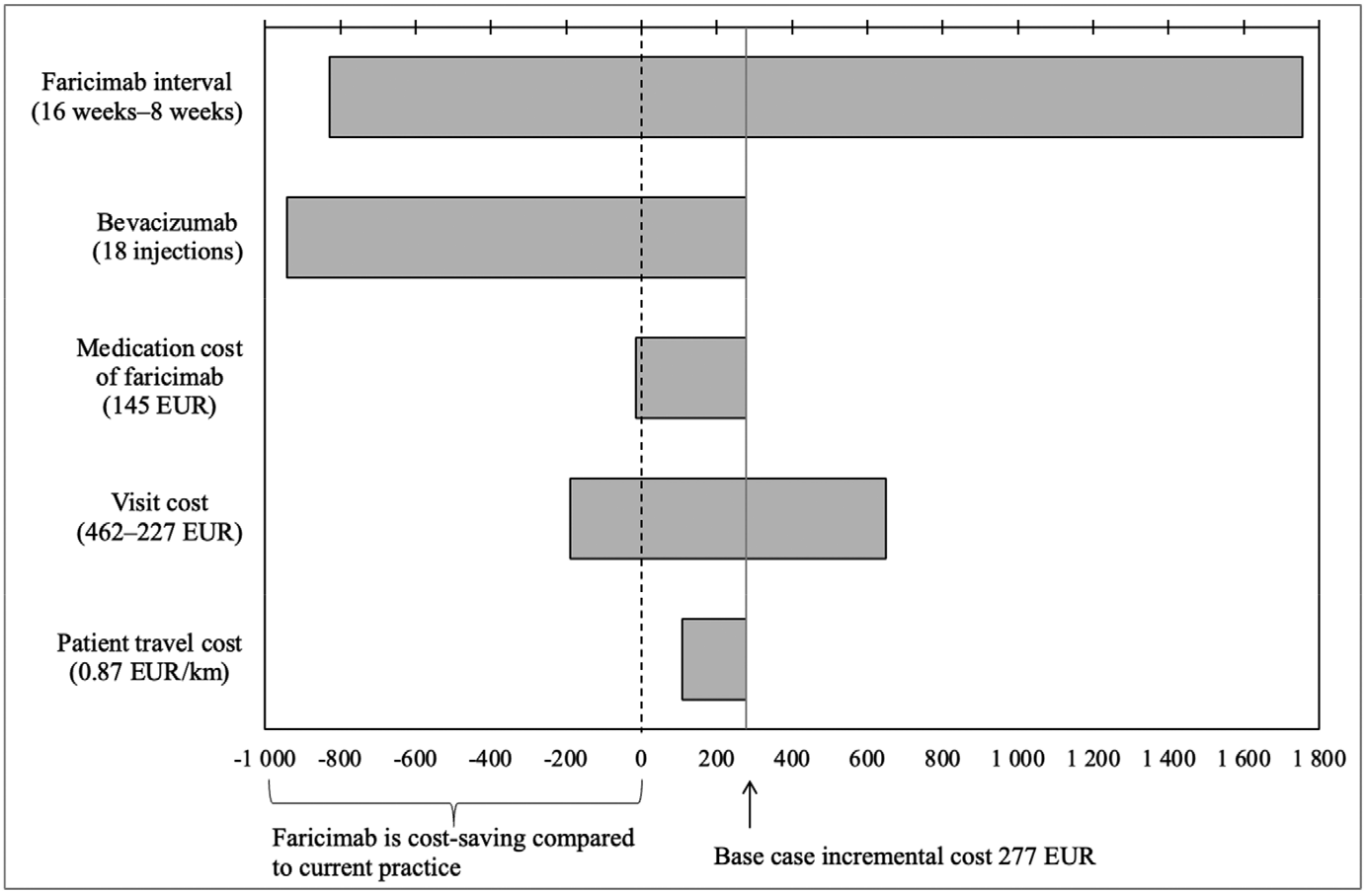
Sensitivity analysis of the incremental societal cost of faricimab per patient in 2023 Euros. Each bar shows the incremental cost when one parameter value is changed with the new parameter value shown in brackets. A positive value means that the incremental cost of faricimab is higher than the current practice whereas a negative value means that faricimab is cost saving.

The sensitivity analysis presented in Figure 3 shows that the injection interval of faricimab and the number of bevacizumab injections have big impacts on the incremental societal cost, that is, the difference in cost per patient between faricimab and current practice over the two‐year period.

If the interval is as high as 16 weeks (6 injections in year 1, and 3 in year 2), faricimab gives a large cost saving (incremental cost −829 EUR per patient). Comparably, with 8‐weeks interval, faricimab is 1755 EUR per patient more expensive compared to current practice. Hence, if other base case parameters are unchanged, a minimum of 12 weeks interval is required for faricimab to be cost saving compared to bevacizumab. If the number of bevacizumab injections is as many as 18 injections over two years, faricimab gives a major cost saving (incremental cost −940 EUR per patient).

It is sufficient to reduce the price of medication by 20% (faricimab medication cost 145 EUR instead of 172 EUR per injection) for faricimab to be cost saving from a societal perspective (−14 EUR per patient). A higher unit cost per visit may also result in cost savings (incremental cost −190 EUR if visit cost is 462 EUR) while a lower visit cost makes faricimab more expensive compared to current practice (incremental cost 650 EUR if visit cost is 227 EUR).

The travel cost per kilometre has a rather small impact on the incremental societal cost. A four‐fold increase reduces the cost difference but faricimab is still more expensive than the current practice (109 EUR if travel cost is valued at 0.87 EUR/km). However, from the patient perspective, this represents a cost saving of 223 EUR per patient compared with current practice.

## 4 DISCUSSION

The results show a similar overall cost between faricimab and the current practice of bevazicumab during the first two years of treatment. Although the total societal cost per patient is slightly higher (by 277 EUR) using faricimab due to the difference in medication costs, savings are made in visit costs and patient travel costs. The main driver of the similar costs is faricimab's ability to prolong the treatment injection interval, thus reducing the number of clinic visits. The societal cost of faricimab is lower the second year (−173 EUR). As nAMD is a chronic disease with several years of injections, cost savings over time can be expected compared to current practice.

A longer treatment interval for nAMD therapy is desirable from both patient and healthcare perspectives. The phase III trials TENAYA and LUCERNE demonstrated that approximately 80% of participants received faricimab every 12 or 16 weeks (Heier et al., 2022; Khanani et al., 2024), indicating that faricimab meets the criteria for an extended treatment interval in trial settings. In our base case scenario, the number of faricimab injections was derived from the results of the TENAYA and LUCERNE trials, resulting in an average reduction of 3.6 visits over the two first years compared to current practice with bevacizumab. We valued this reduction to a saving of 1183 EUR per patient in visit cost to the health providers. In practice, adopting faricimab over bevacizumab would free up time and resources for ophthalmology clinics, allowing healthcare staff to treat more patients with the current workforce. This could potentially lead to reduced waiting times and improved quality of care. Our sensitivity analysis revealed that the incremental cost of faricimab treatment strongly depends on the treatment interval. The average real‐life faricimab treatment interval will be important. If it is 16 weeks, faricimab would lead to large cost savings (−829 EUR) compared to bevacizumab. Even with approximately 12‐week interval, the cost of faricimab was only 277 EUR higher than bevacizumab in the base case analysis, still resulting in 3.6 visits being freed up for that cost difference.

From a societal perspective, it is interesting to investigate how the cost of faricimab compared to bevacizumab varies between different patient groups. Our bevacizumab‐only population, with 14.7 injections over two years, may be a group that is relatively easier to treat compared to the rather large group being excluded (*n* = 437) due to changed treatment from bevacizumab to another anti‐VEGF drug. Two Norwegian studies have shown that an average number of nine injections per year is common among patients with nAMD receiving bevacizumab (Berg et al., 2015; Reitan et al., 2023). Our sensitivity analysis with 18 bevacizumab injections over two years led to cost savings of 940 EUR per patient with faricimab over bevacizumab. In treatment of AMD, various factors affect patients' quality of life, and whether the better‐seeing or worse‐seeing eye is treated plays a crucial role (Jelin et al., 2019). In the study of Reitan et al., 2023, respondents also expressed that every injection was associated with anxiety, stress and struggles to sleep the week prior to the visit. The results of the current study indicate that if faricimab were the first‐line treatment, it would significantly reduce the number of clinical visits thereby lowering costs and stress for both providers and patients.

Many patients in our study population travel long distances to receive their injections, and our results show that faricimab would reduce travel by 26% (257 km) per patient on average over the two‐year period. Among the 25% of patients that travel more than 50 km one‐way, faricimab has almost similar cost (+104 EUR) as current practice from the societal perspective because of the inclusion of patient travel cost. Numerous patients with nAMD, especially those with visual disability or other health issues, experience challenges in travelling independently to clinics (Boyle et al., 2018). Caregivers, such as family members, may therefore need to rearrange their schedules or even take time off from work to accompany the patient. This has broader societal implications, impacting not only the individuals directly involved but also adding to the overall societal cost. It is essential to recognize patient travel cost and caregiver time as valuable resources and include them in economic assessments of different treatments. This study only accounted for the direct patient travel costs that were valued by a fixed price per kilometre equal to the tax‐deductible rate for travelling by own car from the Swedish Tax Agency. Consequently, our results likely underestimate the total societal cost advantage of faricimab, which should be considered when interpreting the results.

The most prominent strength of this study is that it is based on SMR real‐world data from ophthalmology care and therefore uses the exact number of injections of bevacizumab to each participant over the first two years after treatment initiation. Compared to previous health economic evaluations of anti‐VEGF agents by Swedish Dental and Pharmaceutical Benefits Agency (TLV, 2012, 2013, 2020), our cost calculations are both more realistic and more comprehensive. They consider the splitting of ampules and the actual price of bevacizumab rather than the list price, as well as patient travel costs. Since faricimab was not yet utilized in clinical practice during the study period, the assumed number of injections was derived from clinical trials. While TLV reports predominantly focus on patient outcomes in terms of visual acuity, adverse events, and health‐related quality of life, which are not included in the current study, our results provide a more comprehensive understanding of the potential of anti‐VEGF agents to generate cost savings for both providers and patients.

Regarding splitting of ampules, a study performed at Oslo University Hospital investigated the safety of pharmaceutical compounding and splitting of bevacizumab, ranibizumab, and aflibercept ampules into prefilled syringes for intravitreal use considering the risk of post‐injection endophthalmitis (PIE) (Blom et al., 2022). The study found no significant difference in the risk of PIE between procedures using compounded versus clinician‐withdrawn syringes. The results support the safety of pharmaceutical compounding when strict procedures are followed. The current study shows that the medication cost is the primary driving factor for why despite cost savings due to longer treatment intervals (11.1 injections with faricimab versus 14.7 with bevacizumab), faricimab remains the more expensive treatment from both provider and societal perspectives in the base case analysis. The sensitivity analysis showed that a 20% decrease in the price of faricimab would result in a similar societal cost for bevacizumab and faricimab. In our study, the medication cost for faricimab was assumed to be equal to aflibercept. Negotiations with the pharmaceutical company are ongoing in most of the Swedish regions, and the future price of faricimab will have a critical impact on the cost‐effectiveness in ophthalmology care.

### 4.1 Limitations

This health economic evaluation based on data from the SMR has several limitations. In SMR, about a quarter (*n* = 437) of the total number of individuals with nAMD 2017–2022 in the three counties, shifted between treatments at least once during the study period. Shifting between treatments is a significant factor that could impact the decision of which anti‐VEGF agent to use, thereby affecting the relative costs of anti‐VEGF therapies. An analysis that includes shifting between treatments is complicated as several treatments with different prices and treatment intervals would be compared. In addition, it requires assumptions about how shifting patterns observed in the data would be affected by the new treatment. Therefore, only individuals treated with bevacizumab were included in this study. Bevacizumab is also the first‐line treatment in the three counties used in this study, which justifies this decision.

Another complexity was participants getting injections in both eyes during the study period. For some patients that could mean getting injections in both eyes on the same visit while others receive injections on different dates. The provider cost calculation should differentiate between these scenarios so that the cost per visit is lower when both eyes are treated simultaneously. Alternatively, to include only visits where single injections were administered to these patients, manual selection would have been needed, which was considered overly complex and time‐consuming. Therefore, this study only included patients who received treatment in one eye.

Missing data in SMR was another limitation influencing the selection of patients. For some patients, there were gaps between the visits of several months, which could be explained by incomplete data registration in SMR. Therefore, an inclusion criterion was that participants had to undergo treatment throughout the entire study period, and those with incomplete registration were excluded (*n* = 823). Part of the study period also included the Covid‐19 pandemic years. Data from the SMR indicate that the pandemic did not significantly reduce clinic activity (Wickman et al., 2024). However, visits could have been postponed by the patients. Furthermore, since patients are treated using a treat‐and‐extend regimen, visits may not occur precisely at the two‐year mark. The time interval applied to select the study population was therefore two years plus or minus two months. This was implemented to reduce the risk of unintentionally excluding patients from the study population. This did not affect the number of injections each patient received during the two‐year study period, as our analysis accounted for the precise timing at one and two years.

An additional limitation is that the bevacizumab intervals were derived from real‐life data, while the faricimab intervals were from phase III trials. The settings in these populations are different. Our study also includes three regions with likely variation in retreatment criteria, which should be considered when interpreting the results.

### 4.2 Future research

Given that participants in this analysis shifted between treatments, additional cost analyses are warranted to better understand the comparative effectiveness. Specifically, comparisons between faricimab and bevacizumab in this subgroup of patients, as well as with aflibercept, are necessary. Such analyses would enhance our understanding of the most cost‐effective alternative, particularly considering that the medication cost of faricimab was assumed to be equivalent to aflibercept. Further studies are therefore needed for patients shifting between treatments, focusing on identifying the optimal anti‐VEGF therapy for preserving vision.

Faricimab's dual mechanism of action and extended treatment interval hold promise for minimizing challenges associated with changing treatments in nAMD (Kataoka et al., 2024). Recently, aflibercept was approved at a higher dose (8 mg) and extended treatment interval similar to faricimab. Future studies should investigate whether aflibercept or faricimab offer superior health economic benefits.

Future research on faricimab and aflibercept 8 mg, post‐implementation in clinical practice, would be valuable, particularly regarding extended treatment intervals. These studies would provide real‐world data, offering insights into the actual number of injections required and enabling more robust health economic evaluations.

## 5 CONCLUSION

Faricimab represents a cost‐effective alternative to bevacizumab for patients with nAMD in Sweden. Its extended treatment interval is particularly beneficial for patients living far from clinics, and if the real‐life faricimab injection interval extends beyond 12 weeks. Our findings emphasize faricimab's potential to free up healthcare staff to treat a larger patient population with existing clinic resources.

## FUNDING INFORMATION

This study was supported by Umeå University.

## CONFLICT OF INTEREST STATEMENT

SA, A‐M P‐B and HN declare no conflicts of interests. IW is an advisory board member for Bayer and Roche.
